# A Systematic Review on Foster Parents’ Psychological Adjustment and Parenting Style—An Evaluation of Foster Parents and Foster Children Variables

**DOI:** 10.3390/ijerph182010916

**Published:** 2021-10-17

**Authors:** Elisa Mancinelli, Gaia Dell’Arciprete, Silvia Salcuni

**Affiliations:** 1 Department of Socialization and Developmental Psychology, University of Padova, Via Venezia 8, 35121 Padova, Italy; dellarcigaia@gmail.com (G.D.); silvia.salcuni@unipd.it (S.S.); 2 Centre for Foster Care and Family Solidarity (Centro Affidi e Solidarietà Famigliare—Servizi Sociali del Comune di Padova), Via del Carmine 13, 35137 Padova, Italy

**Keywords:** non-relative foster parents, parenting stress, parental distress, parenting style, foster children

## Abstract

The current systematic review aimed to evaluate the variables influencing foster parents’ parenting stress, distress and parenting style, thereby supporting their adjustment and well-being as well as that of foster children. A PRISMA-guided search was conducted in three databases. Observational studies examining parenting stress, parenting distress (subsuming anxiety, depression and stress symptoms) and parenting style—all assessed through validated tools—were considered. A total of 16 studies were included, comprising N = 1794 non-relative foster parents (age range = 30–67 years). Results showed heightened parenting stress over time, both overall and compared to parents at large. Neither foster parents’ nor foster children’s socio-demographic characteristics significantly contributed to the increase in parenting stress; yet child-related stress and children’s externalizing problems were its main predictors. Foster parents’ couple cooperation was associated with reduced parenting stress. Moreover, the authoritative parenting style was associated with parental warmth, while the authoritarian style was associated with foster parents’ greater perceived burden, greater criticism and rejection toward the foster child. Evidence supports the mutual influence between foster parents and children. Foster care services should support foster parents’ needs within a concentric modular system, to ultimately provide better care for both foster parents and children.

## 1. Introduction

Non-relative foster parents are full-time—temporarily—figures providing a safe shelter for children and adolescents within the foster care system [1]. Foster parents take on the responsibility of caring and nurturing them, supporting their psychological adjustment and physical health, as well as ensuring proper schooling and education until they can either be reunited with their birth families, get adopted, or age out of foster care [1,2,3]. Family foster care is generally the preferred option to take care of this youth [4,5,6] and epidemiological data show that, as of 2016, about 790,000 children from industrialized countries, aged between 0 to 17 years, resided in foster care [7]. 

The foster parent role is complex and demanding and includes multi-level stressors that consistently exceed the ordinary challenges that parenthood poses [1,8]. Among the most commonly reported is the complexity of both the child welfare system and its policies and procedures [9,10,11]: Many foster parents report a series of unmet needs and dissatisfaction with children’s agencies, which they often describe as unresponsive to their request, not providing them with appropriate emotional support, financial assistance and proper training and not adequately including them in case planning and management [9,12,13]. A further stressor often reported is the relationship with foster children’s biological families [4,14,15], whose frequently displayed animosity or plain hostility can make scheduled visits highly stressful [15]; foster parents, for their part, might be concerned with the competency of biological parents, especially if their foster children are expected to return under their care, which they often regard as dysfunctional, hence the additional stressor [4,10,15]. In addition to all these challenges, foster children themselves might be an added source of stress [16,17]: As a matter of fact, they come from situations of maltreatment and neglect, and literature shows how early exposure to trauma, abuse and neglect has severe repercussions in several areas leading, for instance, to developmental delays [18], cognitive, emotional and behavioral problems [19,20,21,22], attachment-related difficulties [20] and even chronic medical health problems [18,23]. This, on the whole, identifies foster children as problematic children with special educational needs, particularly challenging to take care of, especially since foster parents often do not feel sufficiently trained to deal with such difficulties [11,24,25]. 

Non-relative foster parents are, though, to be distinguished from kin carers who, albeit playing the same role, present different characteristics and stressors, which must not be disregarded. Kinship caregivers are generally grandparents, aunts, uncles or cousins who take over the role of foster parents for the offspring of immediate or extended family members [26]. Differently from non-relative foster care, kinship care allows the preservation of the child’s blood ties, thus providing children with a more stable family context [27]; this, on the other hand, implies that kin carers need to contain and minimize the contact and negative influence of abusive birth parents upon the child to a greater extent compared to non-relative foster parents [28]. Moreover, from an institutional point of view, they are less closely monitored and regulated by state child-welfare departments than non-relative foster parents; this reflects in a heightened need of standards, guidelines and proper training [28] to help them deal with all the difficulties and issues of foster children, which nonetheless are scarcely provided by the welfare system [29,30]. Lastly, it is noteworthy that, overall, kin carers are configured as an heterogeneous group, whose characteristics and risk factors are more complex to account for [31]. This might limit findings’ generalizability to the broader foster-care population, thereby supporting the need to properly distinguish the population under investigation. 

Everything considered, the experience of being a foster parent can take a toll on individuals’ overall well-being [8,11], putting them “at risk” of experiencing high parenting stress and distress, while also being more likely to suffer from mental and physical health issues [8,25], which might hinder their ability to provide consistent levels of care to children [11]. In this regard, parental distress describes an overall unpleasant emotional experience that parents have in relation to their child and their parental role, and subsumes symptoms of anxiety, depression and stress [32,33,34]. Differently, parenting stress refers to the specific stress that results from parents’ perception of a mismatch between the demands of parenting and their available resources [35,36]. Some studies [37,38,39,40] have highlighted how parenting stress and distress symptoms influence parenting practices and overall parenting style, which is noteworthy considering their relevance for aa foster placement’s success or disruption [4]. While parental practices are the specific behaviors and actions that parents enact when interacting with their children (e.g., helping them do their homework, asking them about their hobbies, etc.), parenting style regards parents’ overall attitude and behavior, which defines the emotional climate in which they raise their children [41,42,43]. Different parenting styles have been defined, with some regarded as less adaptive than others [41,42,43,44]: The authoritative parenting style is considered as the most adaptive and is characterized by clear boundaries and limits set for children by their parents, who nonetheless also display high sensitivity and emotional warmth; differently, the permissive parenting style, although also characterized by high sensitivity and warmth, foresees an indulgent parental behavior and overall lack of discipline, which renders it maladaptive. Further maladaptive parenting styles are the authoritarian and neglectful styles: The former is characterized by strict discipline and high demands but low levels of sensitivity and emotional support, whereas the latter is characteristic of parents who are neither emotionally involved nor set rules and demand discipline from children [41,42,43,44]. Bearing in mind the higher prevalence of psychosocial disturbances of foster children [23,29,45], research studies highlighted how more unfavorable parenting, characterized by harsh or inconsistent punishment and negative control, criticism and rejection, is associated with children’s emotional and behavioral problems [38,39]. In this regard, it is noteworthy that Lamb [46] showed that children’s adjustment is in little to no part determined by the family’s structure or the biological relatedness to parents, but is instead consistently affected by the quality of both parenting and the relationship between parents. As such, differences between foster mothers vs. fathers should be acknowledged, as the foster care situation may be handled and experienced differently by the two, even though most tasks seem equally shared between parental figures [47]. Notwithstanding this, differences in parenting and associated well-being and adjustment have been scarcely investigated within the foster-care context [6,47,48]. 

The intricate interplay between both foster parents’ and children’s behavior and well-being might be better understood through a transactional framework, which accounts for the dynamic processes of mutual influence embedded within families [49]. This specific theoretical framework states that family dynamics are not to be considered unidirectional, but rather bi- or, better yet, multidirectional and on several levels: Characteristics of individual members shape their relationships with others, but dyadic interactions within the family also have an impact on one another and are, in turn, influenced by family and contextual factors. These mutual influences, unfolding over time, continuously determine the specific family setup and functioning and thereby affect the development of children as well [50,51,52]. Consequently, a deeper understanding of the interplay between foster parents and children variables is relevant, as it could provide a more detailed perspective of the foster-care experience. This would, in turn, be an added source of information on which to base the support necessary to favor the well-being of the whole foster family nucleus. Accordingly, Kaasbøll and colleagues [3] argued that having a clearer understanding of all the micro- and macro-processes taking part in such a setting would be useful to develop and implement better trainings and interventions, specifically tailored to the needs and challenges of foster families. This would not only benefit foster parents in terms of specific parenting skills gained and overall enhanced well-being and satisfaction, but is expected to also have a positive impact on foster children’s adjustment and development, increasing chances of a successful placement and, thus, ultimately improving the broader foster-care system [3,8,25].

In light of all this, the current systematic review aims to identify variables associated with foster parents’ psychological adjustment, referring to parenting stress, parental distress (subsuming anxiety, stress and depressive symptoms) and parenting style. The intent is to shed light on which foster parents’ and/or foster children’s variables influence foster parents’ psychological adjustment and parenting style. This would provide insights relevant to adequately support and guide them within their foster parents’ role and, in so doing, promoting foster children’s adjustment and development as well. 

## 2. Materials and Methods

The current systematic review was conducted following the Preferred Reporting Items for Systematic Reviews and Meta-Analyses (PRISMA) guidelines [53]. The related protocol was approved in July 2021 and is available on PROSPERO (Registration Number: CRD42021261657). The PRISMA Checklist [53] is available within the supplementary materials (Table S1).

### 2.1. Eligibility Criteria

#### 2.1.1. Study Design and Characteristics

Observational studies of any design (e.g., cohort studies, case-control studies, case-series studies, cross-sectional studies) that were subject to peer-reviewing, published in academic journals and written either in English or Italian were included; reviews, intervention studies, dissertations, conference abstract, editorials and commentaries were excluded. 

#### 2.1.2. Participants

Eligible participants were non-relative, licensed foster parents; no restrictions were posed for age, gender, ethnicity, sexual orientation or marital status, for foster parents nor for children. Inclusion criteria were intentionally left broad to maximize information regarding the foster-care experience as considered on the whole.

Exclusion criteria were (1) being kinship foster parents; (2) being therapeutic or professional foster parents that had undergone specific and more advanced training; (3) being adoptive parents; (4) being foster parents from group homes; (5) being foster parents of children with disabilities or medical conditions; (6) including non-relative foster parents together with one or more of the abovementioned populations, thus not providing separate information as regards non-relative foster parents vs. the other types of caregivers (see the supplementary materials; Table S2). These exclusion criteria were enacted due to the specificity, in terms of characteristics and stressors, of each of the populations mentioned, which determine the need to differentiate them from the population of interest (i.e., non-relative foster parents).

#### 2.1.3. Outcomes

The outcomes of interest were (1) parenting stress, (2) parental distress (referred to symptoms of anxiety, stress and depression) and (3) parenting style.

Outcomes had to be assessed with validated tools. Studies were excluded if they assessed none of these outcomes or if they did not use validated tools.

### 2.2. Search Strategy

Three electronic databases (i.e., Web of Science, PubMed and PsycINFO) were systematically screened in May 2021 using the following two research keys: (parenting) AND (foster care) AND (stress) AND (distress) AND (anxiety) AND (depression); (parenting) AND (foster care) AND ((stress) OR (distress) OR (anxiety) OR (depression)). Search terms were intentionally left broad to maximize the chances of identifying potentially eligible studies. No search restrictions were posed (e.g., no restrictions in terms of language or publication year, etc.); unpublished studies were not sought.

The title and abstracts of the studies resulting from the electronic search were screened independently by two authors (EM and GDA); potentially eligible studies were then read in full text by the same authors, to assess the fulfillment of the inclusion criteria. The whole process was carried out in a double-blind fashion, and any conflict or discrepancy that occurred in any phase was resolved by discussion or by consulting the third author (SS) until consensus was achieved.

### 2.3. Data Extraction and Management

Data extraction was independently performed by two authors (EM and GDA) and any disagreement was resolved by consulting the third author (SS). The data extracted were the study’s DOI, the first author’s name, publication year, the study’s geographical location, foster parents’ and children’s characteristics (e.g., age, gender, ethnicity, duration of fostering), outcomes of interest and assessment tools used.

### 2.4. Quality Assessment

The methodological quality of the included studies was independently assessed by two authors (EM and GDA) and any conflict was resolved by consulting with the third author (SS). The “Joanna Briggs Institute” (JBI) Critical Appraisal Tools for Systematic Reviews [54] were used. These comprise different checklists based on the specific study design, investigating their methodological quality and the degree to which the study has addressed potential biases through their study design, conduct and analyses performed. Being design specific, different JBI checklists were used according to the design of the included studies. These checklists do not provide a final quality score nor specific criteria to define the overall risk of bias of a study; therefore, judgment on the methodological quality and potential risk of bias of each study was assessed qualitatively, through discussion among the authors, and based on the responses (chosen between “Yes”, “No” and “Unclear”; “Not applicable” was reported where appropriate) given to each question of the checklists investigating the fulfillment of the criteria needed to reduce the studies’ risk of bias (see the supplementary materials; Tables S3–S5).

The JBI Checklists were not employed to assess studies’ eligibility.

## 3. Results

### 3.1. Search Results 

**Figure 1:**
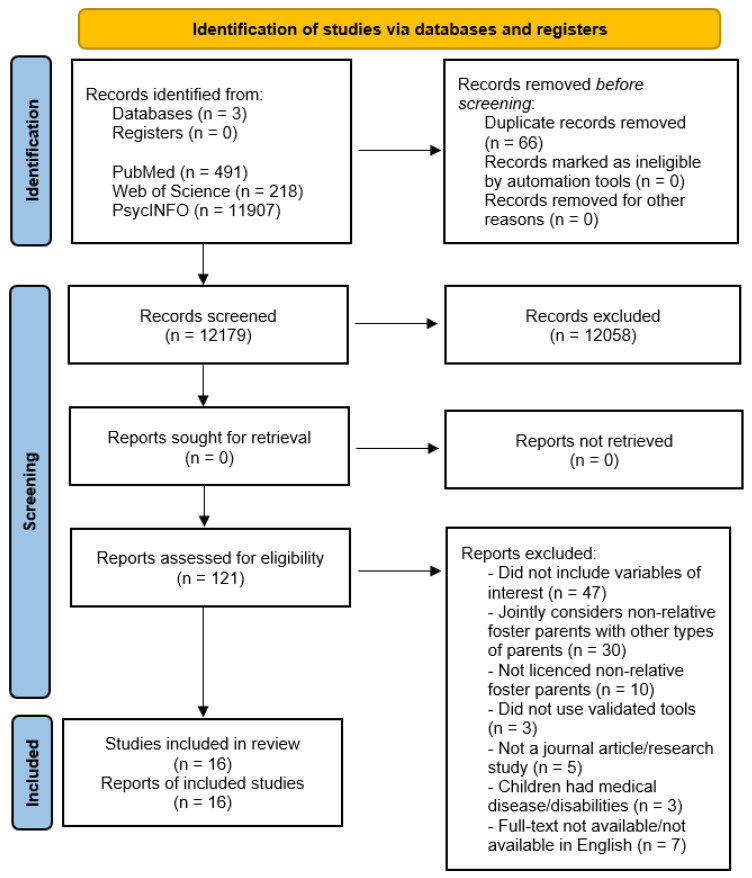
PRISMA 2020 flow diagram.

As shown in Figure 1, the initial database search yielded a total of 12,245 studies. Upon removing duplicates, the first title and abstract screening of 12,179 papers was performed, resulting in a total of 121 studies selected for full-text screening. Then, 105 studies were excluded, in line with inclusion and exclusion criteria, and 16 studies were finally included. Excluded studies, with reasons for exclusion, are reported in the supplementary materials (Table S2).

### 3.2. Studies Characteristics

Studies’ characteristics are reported in Table 1. The included studies were conducted between 2011 and 2020 and comprised a total of N = 1794 non-relative foster parents, whose ages ranged between 30 and 67 years; *n* = 2 studies did not report foster parents’ age [55,56]. Of all the included studies, *n* = 9 included foster mothers [57,58,59,60,61,62,63,64,65] while *n* = 5 [66,67,68,69,70] showed a more homogeneous gender distribution among foster parents; *n* = 2 studies [55,56] did not report information on foster parents’ gender distribution. As shown in Table 1, foster parents were mostly married, albeit *n* = 6 studies [55,56,62,64,68,69] did not report information on foster parents’ marital status. Only two studies [66,67] provided information on foster couples’ sexual orientation.

Around half of the studies [55,56,57,59,60,66,67,68,69] provided information on foster children, showing quite a homogenous gender distribution. Foster children were aged 23 months (i.e., almost 2 years) to 17.8 years. In two studies only [66,67], children were over 10 years of age. The duration of fostering was only reported in *n* = 8 studies [55,56,57,59,60,66,67,68] and ranged from 78.27 days (i.e., around 2 and a half months) to 87.3 months (i.e., over 7 years). Furthermore, *n* = 8 studies [55,56,57,59,60,66,68,69] investigated the effect of foster children’s psychosocial variables on either foster parents’ parenting stress or parental style, specifically focusing on children internalizing and externalizing problems. In this regard, as reported in Table 2, *n* = 13 studies investigated parenting stress, *n* = 1 investigated foster parents’ distress referring to anxiety and depression symptoms and *n* = 2 explored parenting style.

Lastly, *n* = 3 studies [55,57,68] compared non-relative foster parents with biological parents raising their birth children outside the foster care system (hereafter “biological control parents”), one study [61] compared foster parents with kin parents and one study compared foster mothers with both kin carers (all females) and biological control mothers [62]. 

### 3.3. Outcomes’ Assessment Tools

As shown in Table 2, parenting stress was assessed either through the Parenting Stress Index (PSI), its short form (PSI 4th edition Short Form [PSI-4-SF]) [36], the Parental Stress Scale (PSS) [71] or the Parental Stress Questionnaire (PSQ) [72]. Specifically, the PSI [36] is a self-report measure that assesses parents’ perceived parenting stress related to the relationship with their children, aged 1 to 12 years, considering both the levels of parenting stress and its main source. In this regard, there are two main stress sources evaluated through the PSI: The parental domain, which describes parents’ feelings associated with their role and experience as parents beyond the direct relationship with the child, and the child domain, which instead accounts for the child’s problematic behavior and the dysfunctional parent–child relationship. The PSS [71] was developed as an alternative to the PSI, which is indeed quite lengthy (i.e., 120 items); it assesses parenting stress by investigating parents’ feelings towards the positive and negative experiences linked to parenthood, referring to the parent–child relationship. Lastly, the PSQ [72] is another self-report measure that comprises a subscale named parental stress, which accounts for difficulties related to child-rearing and the parent–child relationship; the PSQ comprehends two further sub-scales related to parents perceived social support, both in general and provided by their partner specifically. 

Anxiety and depression symptoms were instead evaluated through the anxiety and depression subscales of the Brief Symptom Inventory (BSI) [73], a brief self-report measure assessing the psychological symptoms experienced during the preceding week. Parenting style was assessed in both the included studies [66,67] through the Rules and Demands Scale (RDS) [74], a self-report measure that evaluates parents’ behavior and distinguishes specifically between the authoritative, authoritarian and permissive parenting style. 

Lastly, the included studies [55,56,57,59,60,66,68,69] investigating the effect of foster children’s psychosocial variables on those of the foster parents’ used either the Strength and Difficulties questionnaire–Parent report (SDQ-P; [75]) [56,57,69] or the Child Behavior Checklist (CBCL; [76]) [55,59,60,66,68]; both tools are parent-reports.

### 3.4. Parenting Style among Foster Parents

Only two of the included studies investigated parenting styles in foster fathers and mothers [66,67], and they both found higher levels of the authoritative parenting style, as compared to the authoritarian and permissive styles. The authoritative parenting style is highly correlated with reduced criticism or rejection and heightened warmth and communication from parents toward the child, as well as with lower foster children’s impulsivity [67] and withdrawal symptoms [66]. Differently, the authoritarian parenting style correlated with a greater perceived burden on the parents’ part, as referring to the fostering situation, and with heightened criticism and rejection toward the child, but it did not correlate with parental warmth and communication [67]. The authoritarian parenting style was also strongly correlated with greater foster children’s behavior problems [66,67] and impulsivity [67]. As regards the permissive parenting style, it was not associated with any of the mentioned foster children nor foster parent variables, but instead only correlated with the other two parenting styles [67]; specifically, it was negatively correlated with the authoritative parenting style and positively correlated with the authoritarian one, albeit showing a small effect size in both cases (i.e., r < 0.20) [67].

In their study, García-Martín and colleagues [67] also attempted to identify at-risk placement profiles, accounting for both foster parents’ and foster children’s variables. Specifically, through k-mean cluster analysis (i.e., a partitional group analysis that allows the subdivision of a set of objects/participants into k groups based on their attributes), the authors identified three profiles, resulting from the differentiation of high, medium and low scores among the following correlated variables: Authoritative parenting styles, parental burden and foster children’s behavioral problems and impulsivity. It is noteworthy that, among the parental variables, the three profiles (high, medium and low “problem groups”) altogether only significantly differed in authoritative parenting style levels, while only the “high problems” profile showed significantly greater burden and criticism compared to the medium and low problems profiles, which instead showed comparable mean scores. 

### 3.5. Parenting Stress and Distress Symptoms

#### 3.5.1. Parenting Stress among Foster Parents—Contextual and Individual Factors

Most of the included studies investigated foster parents’ parenting stress (Table 2), yet the associations between parenting stress and both foster parents and foster children’s socio-demographic data were only marginally assessed [58,59,60,63,68,69]. Results showed no association between parenting stress and foster parents’ age [58,59,60,69] nor with foster children’s age [59,60,68,69] or gender [69]. Moreover, no association was reported between parenting stress and the number of years spent as foster parents, nor with the number of children fostered [58]. No association was found between foster parents’ parenting stress and their occupational status or educational level [69]. As regards foster parents’ economic situation, no study evaluated the association between income and parenting stress, although one study highlighted foster parents that felt no concerns related to their economic situation (described as “I always have money left”) showed significantly lower parenting stress compared to those reporting even slight concerns [63].

Referring to foster parents’ marital status, only one study, conducted during the COVID-19 pandemic, specifically compared married and unmarried (comprising partnered, separated, widowed, divorced and never married) foster parents as regards their parenting stress levels, and showed significantly lower parenting stress among married foster parents [63]. They also reported a lower sense of being overwhelmed related to the fostering situation and its responsibilities, and greater perceived satisfaction with the foster parent role [63]. Moreover, research investigating foster parents’ relationships status and couple cooperation (e.g., married/in a committed relationship vs. single) [63,65,70] in association with foster parents’ parenting stress highlighted that the foster parents’ perceived social support has a protective factor toward increased parenting stress [69]; however, another study did not support such findings [68]. Notwithstanding this, path analysis results [65,70], specifically accounting for the association between the level of cooperation within the parental couple and parenting stress, showed that cooperation (which implies greater perceived helpfulness and shared responsibility in child-rearing between the parental couple) was associated with lower parenting stress [65,70]. Yet, when specifically accounting for gender differences related to the commonalities and interdependence between foster mothers’ and fathers’ parenting stress, actor–partner interdependence model (APIM) results showed that while foster fathers’ parenting stress was influenced both by their perceived level of a cooperating relationship and the foster mother’s stress, foster mothers’ parenting stress was influenced by their perceived level of a cooperating relationship only [70]. Gabler and colleagues [60] did not investigate gender differences in foster parents’ parenting stress but investigated the effect of the partner’s parenting stress on the one experienced by the parent classified as the “main caregiver”, regardless of them being the foster mother or father. Results of the cross-lagged panel analysis (i.e., a statistical analysis that allows one to control the mutual influence between the investigated variables across time) showed that the parenting stress experienced by the main caregiver at 12 months after child placement was significantly influenced by the partner’s stress levels at placement when controlling for the latter’s parenting stress levels at placement.

Beyond the role played by contextual variables such as social support or the responsibilities shared between spouses as regards foster parents’ parenting stress, the role played by individual factors, such as past personal traumatic experiences and resilience, has also been investigated [58]. In detail, the specific influence of past Adverse Childhood Experiences (ACEs, e.g., divorce, childhood abuse, neglect, domestic violence, parents’ substance abuse) on parenting stress [58], while also accounting for foster parents’ resilience, has been assessed. The authors showed that the number of ACEs was not associated with the current parenting stress level and instead highlighted the predictive and protective role of resilience on foster parents’ parenting stress. Specifically, they observed that high resilience resulted as the sole predictor of reduced parenting stress and, accordingly, was also associated with increased satisfaction toward the parental role, even when controlling for past ACEs, foster parents’ age, time as foster parents and the number of children fostered. Moreover, it has also emerged that foster parents who self-reported “excellent mental health” [63] experienced significantly lower parenting stress, felt less overwhelmed by the fostering situation and were more satisfied with the parental role, compared to foster parents who had reported “very good” or “good” mental health. 

#### 3.5.2. Parenting Stress and Distress—Foster Parents Compared to Biological Families and Kin Carers

As already mentioned, the role of foster parents implies additional challenges compared to parents at large, leading them to experience greater parenting stress [55,57,64], while the opposite result can be expected when considering parenting stress and distress experienced by non-relative foster parents vs. kin carers [61]. Accordingly, a recent study showed that foster parents, compared to kin carers, reported lower parenting stress levels, yet also lower satisfaction with their caregiving role [61]. In Harding and colleagues’ [61] study, differences between foster and kin carers were assessed, controlling for their time spent as carers, albeit not for contact with the biological parents of foster children, which was significantly greater among kin carers. Moreover, foster parents had received much greater support in terms of resources, access to services and support from the agency during the child’s stay [61]. Altogether, these differences in resource availability and contact with foster children’s biological parents might account for kin carers’ greater parenting stress compared to the foster parents. However, a study comparing female caregivers (i.e., non-relative foster mothers; biological control mothers; female kin carers) [62] showed that non-relative foster mothers experience significantly lower anxiety and depression symptoms compared to both female kin carers as well as biological control mothers. The finding whereby foster mothers experience fewer parental distress symptoms compared to biological control mothers [62] is in contrast with the greater parenting stress found among foster mothers compared to biological control mothers [55], while being in line with Gabler and colleagues’ [60] results. Specifically, significantly lower parenting stress emerged among foster parents as compared to normative data (i.e., assessed in parents at large), remaining stable throughout 12 months of child placement. However, the sample considered by Gabler et al. [60] had recently acquired the role of a foster parent, leading the authors to suggest that such contradicting results might have been given by a selection bias (i.e., more stressed parents are less likely to be chosen as foster carers) or from a bias resulting from an idealization of the newly acquired parental role, thus showing higher motivation and self-confidence, which dampened the negative impact of the stressors associated with the fostering situation. Nonetheless, in a previous study, the same authors [59] found no association between parenting stress and placement length. 

Contradicting the above-reported results, there is further evidence that foster parents present increased parenting stress compared to biological control parents, both when compared concurrently to normative data from the general population [64] and longitudinally within a one-year timespan [55]. The evidence highlighting greater parenting stress among foster parents showed that such a difference, as outlined herein, was mostly caused by child-related stress rather than the stress resulting from the perception of the caregiving role itself. Bergsund and colleagues [57] longitudinally investigated the parenting stress experienced by foster parents vs. biological control parents, as assessed when children were 2 (T1), 3 (T2) and 8 (T3) years old. Through multiple mixed-effect models, the authors observed that, although foster and biological control parents differed in child-related stress at all time points when children had reached 8 years (T3), only foster parents showed significantly increased parenting stress associated with their parental role. Still, Bergsund et al.’s [57] results point to child-related stress as the main predictor of parenting stress among both foster and biological control parents, although showing a stronger predictive role among foster parents. On the other hand, controlling for children’s age and mental health issues and accounting for gender differences among parents, as reported above, a previous study observed greater parenting stress among foster mothers compared to biological control mothers [55] referring in particular to the stress associated with the caregiving role, thus beyond the parent–child relationship [68]. Differently, whereas foster and biological control fathers showed comparable parenting stress levels when foster children had been with their foster families for slightly longer than a year [68], throughout the second year of foster children’s placement, foster fathers showed higher parenting stress compared to biological control parents [55]. Coherently, Bergsund et al. [57] showed that while biological control parents’ parenting stress did not increase from when children were 2 to when they were 8, it increased among foster parents during the same timeframe [57]. 

### 3.6. Parenting Stress and Parenting Style and Their Association with Foster Children Psychosocial Symptoms

The gathered evidence showed a significant association between foster parents’ parenting stress and children’s psychosocial symptoms [55,56,57,59,60,68,69]. In particular, data from a longitudinal study [59] showed that greater internalizing and externalizing problems among foster children significantly correlated with increased parenting stress, both at placement and six months later. Yet, after controlling for the symptom level at placement, the association between children’s psychosocial symptoms and parenting stress at 6 months from placement was supported only for internalizing problems [59]. Moreover, performing a cross-lagged panel analysis, results showed that parenting stress and parental sensitivity significantly influenced foster children’s internalizing symptoms at placement when controlling for symptom level at placement. However, the opposite direction of this effect was not reported, thus highlighting that children’s internalizing symptoms did not influence foster parents’ parenting stress. Data from the same broad longitudinal project [60] also showed a significant association between parenting stress and children’s externalizing symptoms 12 months after their placement; yet, in line with previous findings [59], externalizing symptoms at placement did not significantly predict parenting stress at 12 months of placement, regardless of controlling for foster children’s age [60]. 

Lohaus and colleagues [55,68], in two separate studies, compared foster parents and biological control parents accounting for gender differences and the influence of children’s psychosocial symptoms on parenting stress. The two studies, which investigated the association between parental and children’s variables concurrently [68] and longitudinally [55], are part of the same broader project and thus share part of their sample. The first assessment occurred over one year from the children’s placement (M = 17.72 months; SD = 8.61) [55,68] while the two subsequent ones took place at 6-month intervals [55]. Regression analysis results [55,68] showed that children’s externalizing problems were the main predictors of both foster and biological control parents’ parenting stress, but findings regarding the effect of children’s internalizing problems on parenting stress were contradictory. In particular, in line with the above-mentioned evidence [59], Lohaus et al. [68] observed that children’s internalizing symptoms did not explain any additional variance related to both foster and biological control parents’ parenting stress, as assessed over about one year of placement; yet, throughout the second year of placement and onwards [55], the authors showed that foster children’s greater internalizing symptoms significantly predicted an increase in parenting stress among foster mothers specifically.

Investigating the time by group effect through cross-lagged panel analysis, Lohaus et al. [55] highlighted the bidirectional influence between children’s internalizing and externalizing symptoms and parenting stress at each time point. However, these bidirectional associations across the three assessment points were observed only among foster parents. In particular, the main finding was that greater foster children’s externalizing symptoms, assessed at the second time point, significantly predicted both foster mothers and foster fathers’ increased parenting stress at the third assessment (i.e., the third year of foster children’s placement), which was not the case for biological control parents. Furthermore, the authors [55] showed that foster mothers’ parenting stress measured about one year from placement (i.e., the first assessment) and foster fathers’ parenting stress levels assessed after about one and a half years from placement (i.e., the second assessment) significantly predicted increased externalizing symptoms among foster children at the third year of placement (i.e., the third assessment). 

Foster children’s internalizing and externalizing symptoms also seem to be associated with foster parents’ parenting style, although only one of the included studies investigated such an association [66]. Specifically, Fuentes and colleagues [66] showed that only the authoritarian parenting style was significantly and positively correlated with both foster children’s internalizing and externalizing symptoms, showing a medium effect size (i.e., r < 0.33, *p* < 0.01; r = 0.38, *p* < 0.01, respectively), as well as with most of the CBCLS sub-scales (i.e., anxiety and depression symptoms, somatic problems, rule-breaking and aggressive behavior; effect sizes ranged between r = 0.25 and r = 0.40). Authoritative and permissive parenting styles were not correlated with either children’s internalizing or externalizing symptoms. 

### 3.7. Quality Assessment of the Included Studies

Three JBI checklists—for cross-sectional, case-control and case-series studies—have been employed for the quality assessment of the included studies, based on their specific design (Table 2). Detailed results of the quality assessment of each of the included studies, determined by whether or not the checklist’s criteria needed to reduce studies’ risk of bias were met, are reported in tabular form within the supplementary materials, comprising all checklist questions and related answers (Tables S3–S5). 

The Checklist for Cross-Sectional studies investigates sample inclusion criteria; the amount of information provided on participants and study setting; validity and reliability of the exposure measure (not applicable for any of the included studies); criteria used to measure the condition (i.e., being a non-relative foster parent); the presence of confounding factors; strategies to deal with confounding factors; validity and reliability of outcome measurement; and the appropriateness of statistical analysis. 

Based on the qualitative critical judgment made on the overall methodological quality of the included cross-sectional studies, the main concerns identified regard inclusion criteria, which were reported only in few studies, and the identification and management of confounding factors, which were either not addressed or unclearly reported. Moreover, the sample characteristics reported were predominantly inconsistent. Nonetheless, most studies performed appropriate statistical analyses and all studies used valid and reliable tools to assess the outcomes of interest. 

The Checklist for Case-Series studies investigates sample inclusion criteria; the reliability of the condition measurement (not applicable); the validity of the methods identifying the condition (not applicable); consecutive inclusion of participants (regarding the specificity of the inclusion process and time-line); completeness of the participant inclusion process; reporting of the demographic information; reporting of the clinical information; clarity of outcomes and the reporting of follow-up results; reporting of the presenting site(s)/clinic(s) demographic information; and the appropriateness of statistical analysis.

As per the above, the overall judgment made on the methodological quality of the two longitudinal case-series studies included raised some concerns, as neither inclusion criteria nor the site information was clearly reported, thus it was unclear whether the study had thoroughly explained participants’ inclusion process. However, overall sample characteristics were provided, statistical analyses were appropriate, and outcomes were reported. 

The Checklist for Case-Control studies investigates the comparability of the groups being compared; the appropriateness of case-control matching; criteria for identifying cases and controls; validity and reliability of exposure measures (not applicable); the comparability of exposure measures between cases and controls (not applicable); the presence of confounding factors; strategies employed to deal with confounding factors; the validity and reliability of outcome assessment between cases and controls; the appropriateness of the exposure period length; and the appropriateness of statistical analysis.

Base on the overall judgment made, one of the two longitudinal case-control studies included raised some concerns [57] and the other [55] might be at high risk of bias since the majority of the evaluated criteria resulted as unclear or were not satisfied. Specifically, while in Bergsund and colleagues’ study [57] it was clear that groups were comparable, it was unclear in Lohaus and colleagues’ work [55]. Notwithstanding, in both studies, it was unclear or not reported whether the two groups being compared had been appropriately matched or if participants were included using the same criteria; moreover, Lohaus and colleagues [55] had identified confounding factors, which was unclear in Bergsund and colleagues’ work [57]. Neither of the two studies appropriately stated if and how they dealt with confounding factors. Still, both studies adequately assessed their outcomes of interest and the “exposure time” (i.e., if the child placement within the foster family had taken place from a sufficient time), and statistical analyses seemed suitable as well. 

Taking into consideration both the results from the JBI tools (i.e., the fulfillment or non-fulfillment of the checklists’ criteria for each included study) and the following discussion among the authors of the current systematic review, it seems that, overall, the included studies have sufficiently satisfactory methodological quality and are not at high risk of bias; nonetheless, they raise some concerns on their overall quality, which, as outlined above, seems mostly undermined by their poor reporting of information.

## 4. Discussion

The current systematic review aimed to evaluate the variables contributing to foster parents’ psychological adjustment (i.e., parenting stress and parental distress) and parenting style, with the intent of gaining insights relevant to provide psychosocial support to foster parents, with implications on the foster child’s well-being and adjustment as well. To the authors’ knowledge, this is the first systematic review specifically focused on parenting stress, parental distress and parenting style among non-relative foster parents that also evaluates both foster parents’ variables and the influence of foster children’s demographic and psychosocial variables on foster parents’ adjustment and parenting style. 

It is noteworthy that, among the included studies, only one [62] investigated distress variables (i.e., anxiety and depression symptoms), while most investigated foster parents’ parenting stress. In this regard, the evidence that emerged showed increased parenting stress over time among foster parents, both in general and compared to parents at large. Notably, findings highlighted child-related stress as the main source of parenting stress, as related to parent–child dysfunctional interactions as well as to children’s problem behavior. The reviewed evidence has also shown the significant influence of foster children’s psychosocial problems (i.e., internalizing and externalizing problems) on foster parents’ parenting stress, particularly highlighting the predictive role of children’s externalizing problems on overt and more aggressive behavior [77]. Foster children’s internalizing problems, such as withdrawal, and anxiety and depression symptoms [77], although showing an association with foster parents’ parenting stress, were, in general, less investigated compared to the externalizing problems [55,57,60,68], and findings were contradictory [55,59]. Nonetheless, it is noteworthy that children’s internalizing and externalizing problems were all assessed through parents’ reports; thus, they represent foster parents’ perception of foster children’s psychosocial problems. As such, it might be that it is the parental perception of the children’s disturbances, rather than the “objective” children’s psychosocial symptoms, that influences parents’ parenting stress and subsequent behavior. This, in turn, might further justify the greater influence of children’s externalizing symptoms compared to internalizing symptoms. The idea that foster parents’ parenting stress is influenced by their perception of children’s psychosocial problems is also supported by findings regarding the SDQ Parent version [75], which highlight how this tool can work as a proxy measure of foster parents’ parenting stress as assessed through the PSI-SF [36,56].

As regards parenting style, in line with the broader literature [78,79,80,81], the gathered evidence showed that the authoritative parenting style was associated with greater parental warmth and acceptance, as well as with better children’s outcomes, such as reduced withdrawal symptoms and reduced problem behavior [67,78,79,82]. Parents adopting an authoritative parenting style usually display a more collaborative approach with children, as they tailor expectations on the children’s characteristics [78,81]. This indeed favors children’s greater regulation capacities, in terms of greater anger regulation and prosocial behavior [83], which is noteworthy considering that children within the foster care system usually present greater emotional disturbances [45,52,57,84,85]. Still, evidence was contradicting, as Fuentes et al. [66] did not find an association between the authoritative parenting style and children’s psychosocial problems. Findings also stressed the strong and unfavorable influence of an authoritarian parenting style upon both foster parents’ and foster children’s adjustment [66,67,78,79,82], showing it to be associated with children’s increased internalizing and externalizing problems [66]. The association between the authoritarian parenting style and foster parents’ increased perceived burden [67] is also relevant, since, as reported above, foster parents show heightened parenting stress and need to face many stressors because of their fostering role. 

Contextualizing the above-discussed findings within the transactional model of family dynamics [49], it should also be noted that increased foster parents’ parenting stress was indeed associated with increased psychosocial symptoms on the part of the foster child as well [57,59,69]. As such, it might be that when foster parents experience greater parenting stress, they become more authoritarian in an attempt to maintain a sense of control over the situation, but this has consequences for the child’s adjustment [86,87]. This is in line with evidence whereby foster parents experiencing strain are less attentive to children’s mental health and educational needs [16], showing particularly reduced parental abilities when children exhibit externalizing problems [37]. Parenting stress is indeed also associated with parental efficacy: They bi-directionally influence each other and are thought to generate from the same context, and are both associated with children’s behaviors, characteristics and relational qualities [40]. As such, it seems pivotal to provide foster parents with adequate training [3,88] for them to properly acquire and adaptively handle their role as foster parents, while concurrently providing them psychosocial support to reduce their parenting stress, thus buffering its consequences upon their parenting behaviors and attitudes in terms of their parenting style [66,67]. A recent meta-analysis [88] showed interventions’ efficacy in improving foster parents’ sensitivity, parenting attitude and dysfunctional discipline, as well as in reducing their parenting stress and children’s problem behaviors. Nonetheless, no significant effect was shown on children’s attachment security [88]. Moreover, there still seems to be no specific program that has shown superiority compared to others [89].

Overall, evidence from the current systematic review serves to further stress the importance and need to properly train foster parents and to support their psychosocial adjustment [3,88], as it may have repercussions on their parenting behaviors and style in general, with consequences on children’s adjustment and mental health as well [40,49,55,57,59,66,67]. Moreover, no association was found between either foster parents or foster children’s socio-demographic information and foster parents’ parenting stress, thereby suggesting and further underlining that child-related stress is indeed the greatest source of parenting stress overall. Notwithstanding, some protective factors relevant to better and further sustaining foster parents have emerged. In particular, when faced with less contextual stressors, such as having no economic concerns [63] or receiving more resources and support from the fostering services [61], foster parents reported reduced parenting stress. As such, foster care services should be attentive to being responsive to foster parents’ needs [3,64,65]. For instance, foster parents might benefit from greater monitoring of their interactions with foster children’s biological parents or from greater guidance in handling difficult situations with their foster children. This would allow the formation of a concentric modular support system. In more detail, it would favor the development of a concentric system, figuratively similar to a matryoshka, in which the fostering services function as “containers”, supporting foster parents’ practical and psychosocial needs. This would create a protective context around them, setting the bases to allow foster parents to properly care for children, functioning themselves as further “containers” for foster children’s needs, and possibly even for foster children’s birth parents [6,90]. Complementarily, the modular structure of such a system would allow and support the overall system’s cohesion and flexibility, thereby favoring the communication between the parts involved while buffering the effect of changes that might occur regarding the fostering project and the professional figures involved in it.

It is worth noting that being in a committed relationship and showing cooperation within the parental couple [63,65,70] was associated with reduced parenting stress. Couples’ cooperation allows the sharing of the burden and difficulties associated with the whole fostering situation as well as that associated with the rearing and supporting of foster children, who are likely to not only have already faced parenting challenges but also show increased emotional and psychosocial difficulties [45,52,57,84,85]. The reviewed literature does not allow one to draw sound conclusions on the differences between foster mothers and fathers; nonetheless, the general trend suggests that, although the parenting stress experienced by a partner might somewhat influence the other’s [60,70], foster parents seemed mostly self-influenced [4,70]. In this regard, interventions might need to be multi-level, to support greater and more harmonious cooperation within the parental couple [65] while sustaining the individual parent’s resources in parallel, beyond the parental couple. Indeed, one of the reviewed studies emphasized the protective role of resilience toward increased stress, further buffering the effect of past stressors and trauma upon current parenting stress levels [58]. This aspect is relevant regardless of foster parents’ relationship status since interventions fostering resiliency can be at the individual level [91] as well as at the family level [91,92,93]. In this regard, within a “family resilience perspective”, resilience interventions would support the overall coping capacities and adaptation of the family, favoring more positive and adaptive family dynamics as well as greater foster parents’ and children’s adjustment [92,93].

Notwithstanding, the reviewed literature should be interpreted with caution in light of the under-reporting of information that emerged from the quality assessment. As such, future studies should be attentive in thoroughly providing all necessary information, to allow studies’ replicability and reliability of findings. Because of this shared limit, together with the lack of investigation of parental distress and parenting style as compared to parenting stress, compelling conclusions cannot be drawn, particularly regarding the association between variables. Two of the included studies [65,70] specifically present a problematic aspect, which qualifies as a limit of the current review itself and, therefore, must be addressed. Namely, neither of these studies clearly stated if the sample was specific to non-relative foster parents (thus excluding kin carers) or not: While one [65] provided, in general, no information in this regard, the other [70] only addressed it by mentioning it among its limitations. Albeit studies that explicitly stated the inclusion of both non-relative and kin carers were excluded during the search process, the authors of the current systematic review chose to include these two studies, given that the absence of this information was not a clear non-compliance of inclusion criteria [65,70]. Besides, they could still be useful for investigating the interdependence of foster parents considered as a mutually influencing dyad, therefore contributing to provide a useful suggestion for future research.

Indeed, notwithstanding these limitations, findings that emerged from this review, along with their shortcomings, are useful to guide future studies. Notably, future research should be careful to properly account for and investigate the influence of socio-demographic information of foster parents and children upon their adjustment. Non-relative and kin carers should be more carefully distinguished, thereby addressing the differences between the two [61,62]. Moreover, since none of the reviewed studies considered siblings, future research should further investigate the whole foster family adjustment, possibly within a transactional framework, to properly account for the processes underlining family dynamics [49]. Lastly, the role played by institutions, and that of the foster care services directly in contact with the foster families and child, should be greatly accounted for and further investigated.

## 5. Conclusions

To conclude, the current systematic review supports the bi-directional association between foster parents’ and children’s psychosocial adjustment, in line with the transactional model of family dynamics whereby there are “mutual influence processes within families” [49] (p. 192). Notably, child-related stress and children’s externalizing problems emerged as the main predictors of increased foster parents’ parenting stress. Moreover, the authoritative parenting style was the most prevalent among the considered parenting styles, which is noteworthy considering the favorable implications of adopting an authoritative parenting style on children’s adjustment and the unfavorable implications associated with the authoritarian one.

Studies’ shortcomings have been highlighted and relevant insights have emerged, thus providing suggestions useful to guide future research and to develop interventions aimed at supporting both foster parents’ and children’s well-being. Foster care services should be attentive and responsive to foster parents’ needs, thereby supporting them in providing care and support to foster children, within a “matryoshka-like” system.

## Figures and Tables

**Table 1:** Studies’ characteristics.

		Foster Parents Characteristics						Foster Children Characteristics			
Study	Study Location	N	Age M (SD) Range	Gender (%)	Ethnicity (%)	Educational Level (%)	Relationship Status (%)	N	Age M (SD) Range	Gender (%)	Duration of Fostering M (SD) Range
[57] *	Norway	60	FM: 37.8 (5.4) FF: 39.7 (5.2)	FM: 91.7% FF: 8.3%	FM: 91.7% Norwegian 8.3% Other FF: 96.7% Norwegian 3.3% other	54.2% at least two years of full-time higher education 45.8% secondary school or below	80% Married 20% Cohabit	60	23.3 months (0.7)	F: 40% M: 60%	87.3 months (6.0) 74–98
[58]	USA	150	37.74 (9.5)	FM: 93.3% FF: 1.3% n.r.: 5.3%	86% White 1.3% Black/African American 3.3% Hispanic/Latino 1.3% Native American/Alaskan Native 0.7% Asian 1.3% Other/Undisclosed 6% Missing	n.r.	74.7% Married 12% Single 0.7% Widowed 4.7% Divorced 2.7% Partnered 5.3% Missing data	n.a.	n.a.	n.a.	n.a.
[66]	Spain	157	FM: 47.9 (6.8) 29.9–66.3 FF: 46.6 (6.5) 31.4–65.1	FM: 54.8% FF: 45.2%	White European (% n.r.)	33.1% higher degree 30% secondary education 31.8% primary education 5.1% no formal schooling	80.2% Heterosexual couples 4.7% Homosexual couples 15.1% Single parents (all F)	104	11 years (3.2) 5–17.8	F: 46.2% M: 53.8%	3.73 years (2.5)
[59] *	Germany	48	41 (5.6)	FM: 89.58% FF: 10.42%	n.r.	>80% at least an intermediate school leaving certificate (GCE)	92% Married or committed 4% Single 4% Divorced	48	30.60 months (17.29) 9–66	F: 50% M: 50%	81.87 days (38.14)
[60] *	Germany	55	FM: 41.18 (6.01) 26–56 FF: n.r.	FM: 87.3% FF: 12.7%	n.r.	76.4% FM and >84.9% FF had at least a medium school graduation	92.7% Married or committed 3.6% Single 3.6% Divorced	55	33.35 months (18.71) 9–79	F: 50.9% M: 49.1%	78.27 days (37.60)
[67]	Spain	157	FM: 47.9 (6.8) 29.9–66,3 FF: 46.6 (6.5) 31.4–65.1	FM: 54.8% FF: 45.2%	White european (% n.r.)	33.1% higher degree 30% secondary education 31.8% primary education 5.1% no formal schooling	80.2% Heterosexual couples 4.7% Homosexual couples 15.1% Single parents (all F)	104	11 years (3.2) 5–17.8	F: 46.2% M: 53.8%	3.73 years (2.5)
[61]	Australia	210	24.9% <40 39.7% 41–50 35.4% >50	FM: 71.3% FF: 28.1%	n.r.	n.r.	56.1% Married 30.7% Single 13.2% Defacto	n.r.	7 years (n.r.) 6–16	n.r.	n.r.
[68]	Germany	n.r.	FM: 40.54 (6.81) FF: 44.01 (6.73)	n.r.	n.r.	n.r.	n.r.	79	3.49 years (1.32) 2–7	F: 49.4% M: 50.6%	17.72 months (8.61)
[55] *	Germany	179	n.r.	FM: 47.5% FF: 52.5%	n.r.	n.r.	n.r.	94	3.80 years (1.56) 2–7	n.r.	17.63 months (n.r.) 2–24 with few exceptions
[56]	UK	16	n.r.	n.r.	n.r.	n.r.	n.r.	16	n.r. (n.r.) 9–14	n.r.	n.r.
[69]	Egypt	147	FM: 44 (n.r.) 26–65 FF: 56 (n.r.) 32–71	FM: 53.1% FF: 46.9%	n.r.	n.r.	n.r.	78	9.75 years (n.r.) 5–15	F: 68% M: 32%	n.r.
[62]	USA	51	48.4 (13.3)	FM: 100%	49% African American 2% White 45% Latino 4% Biracial	62.8% with more than high school	n.r.	n.a.	n.a.	n.a.	n.a.
[63]	USA	990	41.94 (10.61)	FM: 82.2% FF: 17.1%	89.8% White non-Hispanic 7.6% Black non-Hispanic 0.6% American Indian/Alaskan Native 0.6% Asian/Pacific Islander 1.1% Hispanic	35.1% High school diploma or GED 19.2% Associates 22.1% Bachelor’s degree 19.6% Master’s degree 3.1% Ph.D. 0.8% no educational degree	82.2% Married 17.5% Partnered/separated/widowed/divorced/never married	n.a.	n.a.	n.a.	n.a.
[64]	New Zealand	17	57 (n.r.) 39–71	FM: 82.4% FF: 17.6%	58.8% New Zealand European 29.4% Maori 11.8% other Pacific ethnicity	n.r.	n.r.	n.a.	n.a.	n.a.	n.a.
[65]	USA	59	44.93 (10.26)	FM: 100%	69.5% Caucasian 30.5% n.r.	64.3% completed at least 2 years of postsecondary education 35.7% n.r.	91.5% Married 8.5% Committed	n.a.	n.a.	n.a.	n.a.
[70]	USA	192	43.5 (10.2)	FM: 50% FF: 50%	62.9% Caucasian/Hispanic 37.1% n.r.	71.2% completed or at least attended some college 28.8% n.r.	100% Married	n.a.	n.a.	n.a.	n.a.

Note. * Longitudinal study, whereby all descriptive information reported were collected at baseline; n.r. = not reported; n.a. = not applicable; F = females; M = males; FM = foster mothers; FF = foster fathers; FC = foster children; GCE = General Certificate of Education; GED = General Educational Development.

**Table 2:** Results summary.

Study	Study Design	Variables of Interest	Measurement Tool	Results
[57]	Longitudinal study #	Parenting Stress	PSI	- Compared to biological parents, foster parents showed significantly greater overall parenting stress, and child-related parenting stress. - Over time, foster parents showed an increase in overall parenting stress, parent functioning-related and child-related parenting stress.
[58]	Cross-sectional study	Parenting Stress	PSS	- Greater parental resilience predicted reduced parenting stress. - ACEs no longer predicted parenting stress when accounting for parental resilience’s effect. - No significant differences in parenting stress based on ACEs.
[66]	Cross-sectional study	Parenting Style	RDS	- The greatest percentage of parents showed an authoritarian parenting style. - All CBCL subscales, to the exception of child withdrawal, were associated with greater authoritarian parenting. - Foster children’s withdrawal symptoms were favored by higher authoritative parenting.
[59]	Longitudinal study §	Parenting Stress	PSI	- Parenting stress was not associated with parental sensitivity, nor foster parents’ or foster children’s age. - Greater foster children’s attachment security longitudinally (from placement to 6 months after placement) is associated with reduced parenting stress. - After controlling for children’s symptoms at placement, internalizing symptoms, but not the externalizing ones, longitudinally associated with parenting stress
[60]	Longitudinal study §	Parenting Stress	PSI	- Parenting stress remained stable throughout time. - Foster children’s behavioral problems at 12 months after placement significantly predicted parenting stress. - Partner stress longitudinally predicted parenting stress. - Parenting stress, parental sensitivity and foster parents’ hostility toward the foster child were associated after 12 months of placement.
[67]	Cross-sectional study	Parenting Style	RDS	- Authoritative parenting is associated with greater foster parents’ warmth and proper communication capacities and with reduced foster children’s impulsivity/inattention and foster parents’ criticism and rejection toward the foster child. - Authoritarian parenting was associated with greater foster children’s behavioral problems, impulsivity/inattention, greater perceived burden and with greater criticism and rejective behaviors toward the foster child. - Permissive parenting is positively associated with authoritarian parenting, while negatively with authoritative parenting style.
[61]	Cross-sectional study	Parenting Stress	PSI	- Foster parents, compared to kinship cares, showed reduced perceived parenting stress.
[68]	Cross-sectional study	Parenting Stress	PSQ *	- Accounting for children internalizing and externalizing problems, no differences emerged between foster vs. biological parents parenting stress. - Children externalizing problems were the greatest predictors of both foster and biological parents’ parenting stress. - Social support did not further account for (foster and biological) maternal and foster fathers’ parenting stress.
[55]	Longitudinal study #	Parenting Stress	PSQ *	- Both foster mothers and fathers showed greater parenting stress compared to biological parents. - Foster children’s externalizing behavior only predicted foster fathers parenting stress.
[56]	Cross-sectional study	Parenting Stress	PSI-SF	- Perceived foster parents’ foster children’s adjustment difficulties associated with increased parenting stress.
[69]	Cross-sectional study	Parenting Stress	PSI-SF	- None of the PSI-SF subscales are associated with foster children’s age or gender. - The parent distress subscale was only associated with reduced perceived social support and foster children reduced prosocial behavior.
[62]	Cross-sectional study	Anxiety & Depression symptoms	BSI	- Foster mothers showed significantly lower anxiety and depression symptoms compared to kin carers and biological control parents.
[63]	Cross-sectional study	Parenting Stress	PSS	- Married foster parents showed lower overall parenting stress compared to unmarried carers. - Foster parents that self-reported satisfactory mental health showed lower parenting stress overall.
[64]	Cross-sectional study	Parenting Stress	PSI	- Foster parents’ parenting stress was mostly given by child-related stress (score was around 2 SD above normative data for the child-domain sub-scale).
[65]	Cross-sectional study	Parenting Stress	PSS	- Foster fathers’ parenting stress is associated with greater mothers’ parenting stress as well as reduced fathers’ martial and relationship quality. - Foster mothers parenting stress is only associated with reduced mothers’ relationship quality.
[70]	Cross-sectional study	Parenting Stress	PSS	- Greater foster mothers’ parenting stress is associated with poor perceived social support and co-parenting relationship quality.

Note. # Case-Control study; § Case-Series Study; ACEs = Aversive Child Experiences; BSI = Brief Symptoms Inventory; CBCL = Child Behavior Checklist; PSI = Parenting Stress Index; PSI-SF = Parenting Stress Index-Short Form; PSQ = Parental Stress Questionnaire; * the study only considered the PSQ’s Parental Stress subscale; PSS = Parental Stress Scale; RDS = Rules and Demands Scale.

## Supplementary Materials

The following supplementary materials are available online at https://www.mdpi.com/article/10.3390/ijerph182010916/s1, Table S1: PRISMA 2020 Checklist; Table S2: List of excluded studies with reasons for exclusion; Table S3: Checklist for cross-sectional studies; Table S4: Checklist for case-series studies; Table S5: Checklist for case-control studies.
