# Supplementary material for: A Systematic Review on Foster Parents’ Psychological Adjustment and Parenting Style—An Evaluation of Foster Parents and Foster Children Variables
Source: Int J Environ Res Public Health. 2021 Oct 17;18(20):10916. doi: 10.3390/ijerph182010916 (PMC8535523; doi:10.3390/ijerph182010916)
Supplement: Supplementary file 1 [file ijerph-18-10916-s001.zip › ijerph-1370576-supplementary.pdf]

Table S1. PRISMA 2020 Checklist

Section and Topic	Item #	Checklist item	Location where item is reported
**TITLE**			
Title	1	Identify the report as a systematic review.	1
**ABSTRACT**			
Abstract	2	See the PRISMA 2020 for Abstracts checklist.	1
**INTRODUCTION**			
Rationale	3	Describe the rationale for the review in the context of existing knowledge.	2-4
Objectives	4	Provide an explicit statement of the objective(s) or question(s) the review addresses.	4
**METHODS**			
Eligibility criteria	5	Specify the inclusion and exclusion criteria for the review and how studies were grouped for the syntheses.	4
Information sources	6	Specify all databases, registers, websites, organisations, reference lists and other sources searched or consulted to identify studies. Specify the date when each source was last searched or consulted.	4
Search strategy	7	Present the full search strategies for all databases, registers and websites, including any filters and limits used.	4-5
Selection process	8	Specify the methods used to decide whether a study met the inclusion criteria of the review, including how many reviewers screened each record and each report retrieved, whether they worked independently, and if applicable, details of automation tools used in the process.	4-5
Data collection process	9	Specify the methods used to collect data from reports, including how many reviewers collected data from each report, whether they worked independently, any processes for obtaining or confirming data from study investigators, and if applicable, details of automation tools used in the process.	5
Data items	10a	List and define all outcomes for which data were sought. Specify whether all results that were compatible with each outcome domain in each study were sought (e.g. for all measures, time points, analyses), and if not, the methods used to decide which results to collect.	5
	10b	List and define all other variables for which data were sought (e.g. participant and intervention characteristics, funding sources). Describe any assumptions made about any missing or unclear information.	5
Study risk of bias assessment	11	Specify the methods used to assess risk of bias in the included studies, including details of the tool(s) used, how many reviewers assessed each study and whether they worked independently, and if applicable, details of automation tools used in the process.	5
Effect measures	12	Specify for each outcome the effect measure(s) (e.g. risk ratio, mean difference) used in the synthesis or presentation of results.	n.a.
Synthesis methods	13a	Describe the processes used to decide which studies were eligible for each synthesis (e.g. tabulating the study intervention characteristics and comparing against the planned groups for each synthesis (item #5)).	n.a.
	13b	Describe any methods required to prepare the data for presentation or synthesis, such as handling of missing summary statistics, or data conversions.	n.a.
	13c	Describe any methods used to tabulate or visually display results of individual studies and syntheses.	n.a.
	13d	Describe any methods used to synthesize results and provide a rationale for the choice(s). If meta-analysis was performed, describe the model(s), method(s) to identify the presence and extent of statistical heterogeneity, and software package(s) used.	n.a.
	13e	Describe any methods used to explore possible causes of heterogeneity among study results (e.g. subgroup analysis, meta-regression).	n.a.
	13f	Describe any sensitivity analyses conducted to assess robustness of the synthesized results.	n.a.
Reporting bias assessment	14	Describe any methods used to assess risk of bias due to missing results in a synthesis (arising from reporting biases).	n.a.

Section and Topic	Item #	Checklist item	Location where item is reported
Certainty assessment	15	Describe any methods used to assess certainty (or confidence) in the body of evidence for an outcome.	n.a.
**RESULTS**			
Study selection	16a	Describe the results of the search and selection process, from the number of records identified in the search to the number of studies included in the review, ideally using a flow diagram.	5 - 6 (Figure 1)
	16b	Cite studies that might appear to meet the inclusion criteria, but which were excluded, and explain why they were excluded.	Table S2 (Supplementary materials)
Study characteristics	17	Cite each included study and present its characteristics.	5, 7-10 (Table 1)
Risk of bias in studies	18	Present assessments of risk of bias for each included study.	11; Table S3, S4 and S5 (Supplementary materials)--
Results of individual studies	19	For all outcomes, present, for each study: (a) summary statistics for each group (where appropriate) and (b) an effect estimate and its precision (e.g. confidence/credible interval), ideally using structured tables or plots.	n.a.
Results of syntheses	20a	For each synthesis, briefly summarise the characteristics and risk of bias among contributing studies.	11-19
	20b	Present results of all statistical syntheses conducted. If meta-analysis was done, present for each the summary estimate and its precision (e.g. confidence/credible interval) and measures of statistical heterogeneity. If comparing groups, describe the direction of the effect.	n.a.
	20c	Present results of all investigations of possible causes of heterogeneity among study results.	n.a.
	20d	Present results of all sensitivity analyses conducted to assess the robustness of the synthesized results.	n.a.
Reporting biases	21	Present assessments of risk of bias due to missing results (arising from reporting biases) for each synthesis assessed.	n.a.
Certainty of evidence	22	Present assessments of certainty (or confidence) in the body of evidence for each outcome assessed.	n.a.
**DISCUSSION**			
Discussion	23a	Provide a general interpretation of the results in the context of other evidence.	19-21
	23b	Discuss any limitations of the evidence included in the review.	21
	23c	Discuss any limitations of the review processes used.	21
	23d	Discuss implications of the results for practice, policy, and future research.	21-22
**OTHER INFORMATION**			
Registration and protocol	24a	Provide registration information for the review, including register name and registration number, or state that the review was not registered.	4
	24b	Indicate where the review protocol can be accessed, or state that a protocol was not prepared.	4
	24c	Describe and explain any amendments to information provided at registration or in the protocol.	n.a.
Support	25	Describe sources of financial or non-financial support for the review, and the role of the funders or sponsors in the review.	22
Competing interests	26	Declare any competing interests of review authors.	22
Availability of data, code and other materials	27	Report which of the following are publicly available and where they can be found: template data collection forms; data extracted from included studies; data used for all analyses; analytic code; any other materials used in the review.	n.a.

Table S2. List of excluded studies with reasons

Author, Year	Exclusion reason
[1]	Does not include any of our variables of interest
[2]	Commentary
[3]	Does not include any of our variables of interest
[4]	Does not consider foster parents, but biological parents and children
[5]	Does not consider foster parents, but biological parents and children
[6]	Does not consider foster parents, but biological parents and children
[7]	Does not consider foster parents, but biological parents and children
[8]	Considers jointly foster and kinship caregivers; does not include any of our variables of interest
[9]	Does not consider foster parents, but biological parents
[10]	Does not consider foster parents
[11]	No full text available
[12]	No full text available
[13]	Does not include any of our variables of interest
[14].	Considers jointly foster and adoptive parents; does not include any of our variables of interest
[15]	Considers people that want to become foster parents, but are not yet foster parents
[16]	Does not include any of our variables of interest
[17]	No full text available
[18]	Does not include any of our variables of interest
[19]	Does not use validated tools
[20]	Does not include any of our variables of interest
[21]	Does not include any of our variables of interest
[22]	Does not include any of our variables of interest
[23]	Does not include any of our variables of interest
[24]	Does not include any of our variables of interest
[25]	No full text available
[26]	Does not include any of our variables of interest
[27]	Considers treatment foster parents
[28]	Child has a medical disease (HIV)

[29]	Considers jointly foster and kinship caregivers; does not include any of our variables of interest
[30]	Considers jointly multiple types of foster cares (i.e., relatives, family, or specialized foster caregivers)
[31]	Does not include any of our variables of interest
[32]	Does not include any of our variables of interest
[33]	No full text available
[34]	Does not include any of our variables of interest
[35]	Does not include any of our variables of interest
[36]	Does not include any of our variables of interest
[37]	Does not include any of our variables of interest
[38]	Does not include any of our variables of interest
[39]	Considers jointly foster and kinship caregivers
[40]	Does not include any of our variables of interest
[41]	Not an observational empirical study
[42]	It is a review
[43]	It is a review
[44]	No full text available
[45]	Does not include any of our variables of interest; full text is available only in French
[46]	Does not include any of our variables of interest
[47]	Does not include any of our variables of interest
[48]	Focuses on children
[49]	Does not use validated tools
[50]	Considers jointly foster and kinship caregivers
[51]	Considers jointly foster and kinship caregivers
[52]	Considers jointly foster and kinship caregivers
[53]	Considers jointly foster and kinship caregivers
[54].	Does not include any of our variables of interest
[55]	Does not include any of our variables of interest
[56]	Considers jointly foster and kinship caregivers
[57]	Considers jointly foster and kinship caregivers
[58]	Considers jointly foster and kinship caregivers
[59]	Does not include any of our variables of interest

[60]	Considers jointly foster and kinship caregivers; does not include any of our variables of interest
[61].	Does not include any of our variables of interest
[62]	Does not include any of our variables of interest
[63]	Does not include any of our variables of interest
[64]	Does not consider foster parents; does not include any of our variables of interest
[65]	Does not use a validated tool
[66]	Does not include any of our variables of interest
[67]	Does not include any of our variables of interest
[68]	Considers jointly foster and kinship caregivers; does not include any of our variables of interest
[69]	Does not include any of our variables of interest
[70]	Does not include any of our variables of interest
[71]	Does not include any of our variables of interest
[72]	Considers jointly foster, specialized and kinship caregivers
[73]	Considers jointly foster and kinship caregivers
[74]	Does not include any of our variables of interest
[75]	Does not include any of our variables of interest
[76]	Considers jointly foster and adoptive caregivers
[77]	Considers jointly foster and kinship caregivers; does not include any of our variables of interest
[78]	Considers jointly foster and kinship caregivers
[79]	Considers jointly foster and kinship caregivers
[80]	Proposes a conceptual model; it is not an observational study
[81]	Does not include any of our variables of interest
[82]	Does not include any of our variables of interest
[83]	Does not include any of our variables of interest
[84]	Considers jointly foster and kinship caregivers
[85]	Considers jointly foster and kinship caregivers
[86]	Considers jointly foster and adoptive parents; does not include any of our variables of interest
[87]	Considers jointly foster and kinship caregivers
[88]	Considers jointly foster and adoptive caregivers
[89]	Does not include any of our variables of interest

[90]	Does not include any of our variables of interest
[91]	Considers drug-exposed children
[92]	Does not consider foster parents
[93]	Full text is available only in German
[94]	Considers jointly foster and kinship caregivers
[95]	Considers jointly foster and kinship caregivers
[96]	Considers jointly foster and kinship caregivers
[97]	Does not include any of our variables of interest
[98]	Considers jointly foster and kinship caregivers
[99]	Considers jointly foster and kinship caregivers
[100]	Considers jointly foster and kinship caregivers
[101]	Considers jointly foster and kinship caregivers
[102]	Considers jointly foster and kinship caregivers
[103]	Considers jointly foster and kinship caregivers
[104]	Does not include any of our variables of interest
[105]	Does not include any of our variables of interest

Table S3. Checklist for Cross-Sectional studies

	[106]	[107]	[108]	[58]	[109]	[80]	[110]	[111]	[112]	[113]	[114]	[115]
Were the criteria for inclusion in the sample clearly defined?	Yes	No	Yes	No	No	No	No	No	Yes	Unclear	Yes	Unclear
Were the study subjects and the setting described in detail?	No	Yes	Yes	No	Yes	Unclear	Yes	No	Yes	Unclear	Yes	Yes
Was the exposure measured in a valid and reliable way?	n.a.	n.a.	n.a.	n.a.	n.a.	n.a.	n.a.	n.a.	n.a.	n.a.	n.a.	n.a.
Were objective, standard criteria used for measurement of the condition?	Unclear	Unclear	Unclear	Unclear	Unclear	Unclear	Yes	Unclear	Yes	Unclear	Yes	Unclear
Were confounding factors identified?	No	No	n.a.	Yes	Yes	No	No	Yes	Unclear	No	No	Yes
Were strategies to deal with confounding factors stated?	Unclear	No	n.a.	No	Yes	n.a.	No	Yes	Unclear	No	No	No
Were the outcomes measured in a valid and reliable way?	Yes	Yes	Yes	Yes	Yes	Yes	Yes	Yes	Yes	Yes	Yes	Yes
Was appropriate statistical analysis used?	Yes	Unclear	Yes	Yes	Yes	Yes	Yes	Yes	Yes	Unclear	Yes	Yes

Note. n.a. = Not applicable

Table S4. Checklist for Case-Series studies

	[116]	[117]
Were there clear criteria for inclusion in the case series?	No	No
Was the condition measured in a standard, reliable way for all participants included in the case series?	n.a.	n.a.
Were valid methods used for identification of the condition for all participants included in the case series?	n.a.	n.a.
Did the case series have consecutive inclusion of participants?	Yes	Yes
Did the case series have complete inclusion of participants?	Unclear	Unclear
Was there clear reporting of the demographics of the participants in the study?	Yes	Yes
Was there clear reporting of clinical information of the participants?	Unclear	Unclear
Were the outcomes or follow up results of cases clearly reported?	Yes	Yes
Was there clear reporting of the presenting site(s)/clinic(s) demographic information?	No	No
Was appropriate statistical analysis used?	Yes	Yes

Note. n.a. = Not applicable

Table S5. Checklist for Case-Control studies

	[118]	[119]
Were the groups comparable other than the presence of disease in cases or the absence of disease in controls?	Yes	Unclear
Were cases and controls matched appropriately?	No	Unclear
Were the same criteria used for identification of cases and controls?	Unclear	Unclear
Was exposure measured in a standard, valid and reliable way?	n.a.	n.a.
Was exposure measured in the same way for cases and controls?	n.a.	n.a.
Were confounding factors identified?	Yes	Unclear
Were strategies to deal with confounding factors stated?	No	No
Were outcomes assessed in a standard, valid and reliable way for cases and controls?	Yes	Yes
Was the exposure period of interest long enough to be meaningful?	Yes	Yes
Was appropriate statistical analysis used?	Yes	Yes

Note. n.a. = Not applicable

Bibliography – Supplementary Materials

1. Ackerman, J.P.; Dozier, M. The influence of foster parent investment on children's representations of self and attachment figures. *J. Appl. Dev. Psychol.* **2005**, *26*, 507–520, doi:10.1016/j.appdev.2005.06.003.

2. Adnopoz, J. Addressing symptoms of depression in foster caregivers may improve the quality of care children receive. *Child Abuse Negl.* **2007**, *31*, 291–293, doi:10.1016/j.chiabu.2007.02.003.

3. Ahn, H.; Greeno, E.J.; Bright, C.L.; Hartzel, S.; Reiman, S. A survival analysis of the length of foster parenting duration and implications for recruitment and retention of foster parents. *Child. Youth Serv. Rev.* **2017**, *79*, 478–484, doi:10.1016/j.childyouth.2017.06.069.

4. Adkins, T.; Luyten, P.; Fonagy, P. Development and Preliminary Evaluation of Family Minds: A Mentalization-based Psychoeducation Program for Foster Parents. *J. Child Fam. Stud.* **2018**, *27*, 2519–2532, doi:10.1007/s10826-018-1080-x.

5. Akin, B.A.; Lang, K.; McDonald, T.P.; Yan, Y.; Little, T. Randomized study of PMTO in foster care: Six-month parent outcomes. *Res. Soc. Work Pract.* **2018**, *28*, 810–826, doi:10.1177/1049731517703746.

6. Akin, B.A.; Lang, K.; Yan, Y.; McDonald, T.P. Randomized trial of PMTO in foster care: 12-month child well-being, parenting, and caregiver functioning outcomes. *Child. Youth Serv. Rev.* **2018**, *95*, 49–63, doi:10.1016/j.childyouth.2018.10.018.

7. Akin, B.A.; McDonald, T.P. Parenting intervention effects on reunification: A randomized trial of PMTO in foster care. *Child Abuse Negl.* **2018**, *83*, 94–105, doi:10.1016/j.chiabu.2018.07.011.

8. Albertson, K.; Crouch, J.M.; Udell, W.; Schimmel-Bristow, A.; Serrano, J.; Ahrens, K.R. Caregiver-endorsed strategies to improving sexual health outcomes among foster youth. *Child Fam. Soc. Work* **2020**, doi:10.1111/cfs.12726.

9. Alpert, L.T.; Britner, P.A. Measuring parent engagement in foster care. *Soc. Work Res.* **2009**, *33*, 135–145, doi:10.1093/swr/33.3.135.

10. Aracena, M.; Gomez, E.; Undurraga, C.; Leiva, L.; Marinkovic, K.; Molina, Y. Validity and Reliability of the Parenting Stress Index Short Form (PSI-SF) Applied to a Chilean Sample. *J. Child Fam. Stud.* **2016**, *25*, 3554–3564, doi:10.1007/s10826-016-0520-8.

11. Argall, N.L.; Johnson, E.; Cox, E.; Hislop, C.; Lefmann, S.A. Exploring parental perceptions of a family-centred model of care in a public child development service. *J. child Heal. care Prof. Work. with Child. Hosp. community* **2021**, 1367493521993972, doi:10.1177/1367493521993972.

12. Aslamazova, L.A.; Yurina, A.A.; Khakunova, F.P.; Kochenkova, L.P. Peculiarities of relationships between foster parents and their foster children with disabilities. *Int. J. Environ. Sci. Educ.* **2016**, *11*, 3353–3366.

13. Ballen, N.; Bernier, A.; Moss, E.; Tarabulsy, G.M.; St-Laurent, D. Insecure attachment states of mind and atypical caregiving behavior among foster mothers. *J. Appl. Dev. Psychol.* **2010**, *31*, 118–125, doi:10.1016/j.appdev.2009.10.001.

14. Barnett, E.R.; Jankowski, M.K.; Butcher, R.L.; Meister, C.; Parton, R.R.; Drake, R.E. Foster and Adoptive Parent Perspectives on Needs and Services: a Mixed Methods Study. *J. Behav. Health Serv. Res.* **2018**, *45*, 74–89, doi:10.1007/s11414-017-9569-4.

15. Baum, A.C.; Crase, S.J.; Crase, K.L. Influences on the decision to become or not become a foster parent. *Fam. Soc.* **2001**, *82*, 202–213, doi:10.1606/1044-3894.205.

16. Bernard, K.; Dozier, M. This is my baby: Foster parents' feelings of commitment and displays of delight. *Infant Ment. Health J.* **2011**, *32*, 251–262, doi:10.1002/imhj.20293.

17. Bilanakis, N.D.; Pappas, E.E.; Lecic-Tosevski, D.; Alexiou, D.B. Children of war fostered by Greek families for six months: The effect of the programme on children and foster mothers by. *Eur. J. Psychiatry* **1999**, *13*, 215–222.

18. Bridger, K.M.; Binder, J.F.; Kellezi, B. Secondary traumatic stress in foster carers: Risk factors and implications for intervention. *J. Child Fam. Stud.* **2020**, *29*, 482–492, doi:10.1007/s10826-019-01668-2.

19. Brown, J.D.; Arnault, D. St.; George, N.; Sintzel, J. Challenges of transcultural placements: Foster parent perspectives. *Child Welf. J. Policy, Pract. Progr.* **2009**, *88*, 103–126.

20. Brown, J.D.; Bednar, L.M. Foster parent perceptions of placement breakdown. *Child. Youth Serv. Rev.* **2006**, *28*, 1497–1511, doi:10.1016/j.childyouth.2006.03.004.

21. Brown, J.D.; Rodgers, J.; Ivanova, V.; Mehta, N.; Skrodzki, D. Mental needs of Aboriginal foster parents. *Child Adolesc. Soc. Work J.* **2014**, *31*, 539–557, doi:10.1007/s10560-014-0335-7.

22. Brown, J.D. Foster parents' perceptions of factors needed for successful foster placements. *J. Child Fam. Stud.* **2008**, *17*, 538–554, doi:10.1007/s10826-007-9172-z.

23. Brown, J.; Calder, P. Concept mapping the needs of foster parents. *Child Welf. J. Policy, Pract. Progr.* **2000**, *79*, 729–746.

24. Brown, J. Fostering children with disabilities: A concept map of parent needs. *Child. Youth Serv. Rev.* **2007**, *29*, 1235–1248, doi:10.1016/j.childyouth.2007.05.009.

25. Brown, J.D.; Rodger, S. Children with disabilities: Problems faced by foster parents. *Child. Youth Serv. Rev.* **2009**, *31*, 40–46, doi:10.1016/j.childyouth.2008.05.007.

26. Buehler, C.; Cox, M.E.; Cuddeback, G. Foster Parents' Perceptions of Factors that Promote or Inhibit Successful Fostering. *Qual. Soc. Work Res. Pract.* **2003**, *2*, 61–83, doi:10.1177/1473325003002001281.

27. Castellanos-Brown, K.; Lee, B. Transitioning foster youth to less restrictive settings: Perspectives of treatment foster parents. *Fam. Soc.* **2010**, *91*, 142–148, doi:10.1606/1044.3894.3973.

28. Chalfin, S.R.; Grus, C.L.; Tomaszeski, L. Caregivers's stress secondary to raising young children with HIV infection: A preliminary investigation. *J. Clin. Psychol. Med. Settings* **2002**, *9*, 211–218, doi:10.1023/A:1016099227461.

29. Chamberlain, P.; Price, J.; Leve, L.D.; Laurent, H.; Landsverk, J.A.; Reid, J.B. Prevention of behavior problems for children in foster care: Outcomes and mediation effects. *Prev. Sci.* **2008**, *9*, 17–27, doi:10.1007/s11121-007-0080-7.

30. Cole, S.A.; Eamon, M.K. Predictors of depressive symptoms among foster caregivers. *Child Abuse Negl.* **2007**, *31*, 295–310, doi:10.1016/j.chiabu.2006.06.010.

31. Colton, M.J. Carers of children: A comparative study of the practices of residential and foster carers. *Child. Soc.* **1992**, *6*, 25–37, doi:10.1111/j.1099-0860.1992.tb00385.x.

32. Combs-Orme, T.; Orme, J.G. Foster parenting together: Assessing foster parent applicant couples. *Child. Youth Serv. Rev.* **2014**, *36*, 70–80, doi:10.1016/j.childyouth.2013.10.017.

33. Cooley, M.E.; Thompson, H.M.; Wojciak, A.S.; Mihalec-Adkins, B.P. Parental monitoring by foster parents, youth behaviours and the youth–foster parent relationship. *Child Fam. Soc. Work* **2021**, doi:10.1111/cfs.12844.

34. Cooley, M.E.; Farineau, H.M.; Mullis, A.K. Child behaviors as a moderator: Examining the relationship between foster parent supports, satisfaction, and intent to continue fostering. *Child Abuse Negl.* **2015**, *45*, 46–56, doi:10.1016/j.chiabu.2015.05.007.

35. Cooley, M.E.; Petren, R.E. Foster parent perceptions of competency: Implications for foster parent training. *Child. Youth Serv. Rev.* **2011**, *33*, 1968–1974, doi:10.1016/j.childyouth.2011.05.023.

36. Cooley, M.E.; Thompson, H.M.; Newell, E. Examining the influence of social support on the relationship between child behavior problems and foster parent satisfaction and challenges. *Child Youth Care Forum* **2019**, *48*, 289–303, doi:10.1007/s10566-018-9478-6.

37. Cooley, M.; Wojciak, A.S.; Farineau, H.; Mullis, A. The association between perception of relationship with caregivers and behaviours of youth in foster care: a child and caregiver perspective. *J. Soc. Work Pract.* **2015**, *29*, 205–221, doi:10.1080/02650533.2014.933405.

38. De Maeyer, S.; Vanderfaeillie, J.; Robberechts, M.; Vanschoonlandt, F.; Van Holen, F. Foster parents' coping style and

attitudes toward parenting. *Child. Youth Serv. Rev.* **2015**, *53*, 70–76, doi:10.1016/j.childyouth.2015.03.023.

39. De Robertis, M.T.; Litrownik, A.J. The experience of foster care: Relationship between foster parent disciplinary approaches and aggression in a sample of young foster children. *Child Maltreat.* **2004**, *9*, 92–102, doi:10.1177/1077559503260402.

40. Dorsey, S.; Pullmann, M.D.; Berliner, L.; Koschmann, E.; McKay, M.; Deblinger, E. Engaging foster parents in treatment: a randomized trial of supplementing trauma-focused cognitive behavioral therapy with evidence-based engagement strategies. *Child Abuse Negl.* **2014**, *38*, 1508–1520, doi:10.1016/j.chiabu.2014.03.020.

41. Douglas, M. Using transactional analysis to help foster parents develop therapeutic parenting skills. *Trans. Anal. J.* **2018**, *48*, 335–349, doi:10.1080/03621537.2018.1505129.

42. Dozier, M.; Albus, K.; Fisher, P.A.; Sepulveda, S. Interventions for foster parents: Implications for developmental theory. *Dev. Psychopathol.* 2002, *14*, 843–860.

43. Dozier, M.; Higley, E.; Albus, K.E.; Nutter, A. Intervening with foster infants' caregivers: Targeting three critical needs. *Infant Ment. Health J.* **2002**, *23*, 541–554, doi:10.1002/imhj.10032.

44. Edelstein, S.B.; Burge, D.; Waterman, J. Helping foster parents cope with separation, loss, and grief. *Child Welf. J. Policy, Pract. Progr.* **2001**, *80*, 5–25.

45. Euillet, S.; Zaouche-Gaudron, C. Assistants familiaux: Une parentalité d'accueil? = Foster parents: Foster parenting? *Prat. Psychol.* **2007**, *13*, 365–375, doi:10.1016/j.prps.2007.06.005.

46. Farmer, E.; Lipscombe, J.; Moyers, S. Foster Carer Strain and its Impact on Parenting and Placement Outcomes for Adolescents. *Br. J. Soc. Work* **2005**, *35*, 237–253, doi:10.1093/bjsw/bch181.

47. Fawley-King, K.; Trask, E. V; Ferrand, J.; Aarons, G.A. Caregiver strain among biological, foster, and adoptive caregivers caring for youth receiving outpatient care in a public mental health system. *Child. Youth Serv. Rev.* **2020**, *111*, doi:10.1016/j.childyouth.2020.104874.

48. Fisher, P.A.; Gunnar, M.R.; Chamberlain, P.; Reid, J.B. Preventive intervention for maltreated preschool children: impact on children's behavior, neuroendocrine activity, and foster parent functioning. *J. Am. Acad. Child Adolesc. Psychiatry* **2000**, *39*, 1356–1364, doi:10.1097/00004583-200011000-00009.

49. Garcia, A.; O'Reilly, A.; Matone, M.; Kim, M.; Long, J.; Rubin, D.M. The influence of caregiver depression on children in non-relative foster care versus kinship care placements. *Matern. Child Health J.* **2015**, *19*, 459–467, doi:10.1007/s10995-014-1525-9.

50. Goemans, A.; Geel, M. van; Vedder, P. Foster children's behavioral development and foster parent stress: testing a transactional model. *J. Child Fam. Stud.* **2018**, *27*, 990–1001, doi:10.1007/s10826-017-0941-z.

51. Goemans, A.; Buisman, R.S.M.; van Geel, M.; Vedder, P. Foster parent stress as key factor relating to foster children's mental health: A 1-year prospective longitudinal study. *Child Youth Care Forum* **2020**, *49*, 661–686, doi:10.1007/s10566-020-09547-4.

52. Greeno, E.J.; Lee, B.R.; Uretsky, M.C.; Moore, J.E.; Barth, R.P.; Shaw, T. V Effects of a foster parent training intervention on child behavior, caregiver stress, and parenting style. *J. Child Fam. Stud.* **2016**, *25*, 1991–2000, doi:10.1007/s10826-015-0357-6.

53. Greeno, E.J.; Uretsky, M.C.; Lee, B.R.; Moore, J.E.; Barth, R.P.; Shaw, T. V Replication of the KEEP foster and kinship parent training program for youth with externalizing behaviors. *Child. Youth Serv. Rev.* **2016**, *61*, 75–82, doi:10.1016/j.childyouth.2015.12.003.

54. Haight, W.L.; Black, J.E.; Mangelsdorf, S.; Giorgio, G.; Tata, L.; Schoppe, S.J.; Szewczyk, M. Making visits better: the perspectives of parents, foster parents, and child welfare workers. *Child Welfare* **2002**, *81*, 173–202.

55. Hannah, B.; Woolgar, M. Secondary trauma and compassion fatigue in foster carers. *Clin. Child Psychol. Psychiatry* **2018**, *23*, 629–643, doi:10.1177/1359104518778327.

56. Harden, B.J.; Meisch, A.D.; Vick, J.E.; Pandohie-Johnson, L. Measuring parenting among foster families: The development of the Foster Parent Attitudes Questionnaire (FPAQ). *Child. Youth Serv. Rev.* **2008**, *30*, 879–892, doi:10.1016/j.childyouth.2007.12.015.

57. Harding, L.; Murray, K.; Shakespeare-Finch, J.; Frey, R. High stress experienced in the foster and kin carer role: Understanding the complexities of the carer and child in context. *Child. Youth Serv. Rev.* **2018**, *95*, 316–326, doi:10.1016/j.childyouth.2018.11.004.

58. Harding, L.; Murray, K.; Shakespeare-Finch, J.; Frey, R. The wellbeing of foster and kin carers: A comparative study. *Child. Youth Serv. Rev.* **2020**, *108*, doi:10.1016/j.childyouth.2019.104566.

59. Holtan, A.; Handegård, B.H.; Thørnblad, R.; Vis, S.A. Placement disruption in long-term kinship and nonkinship foster care. *Child. Youth Serv. Rev.* **2013**, *35*, 1087–1094, doi:10.1016/j.childyouth.2013.04.022.

60. Jacobsen, H.; Brabrand, H.; Liland, S.M.M.; Wentzel-Larsen, T.; Moe, V. Foster parents' emotional investment and their young foster children's socio-emotional functioning. *Child. Youth Serv. Rev.* **2018**, *86*, 200–208, doi:10.1016/j.childyouth.2018.01.020.

61. Jiménez-Morago, J.M.; León, E.; Algeciras, C. Parental sense of competence among non-kin foster carers from Spain. *Child. Youth Serv. Rev.* **2018**, *94*, 437–445, doi:10.1016/j.childyouth.2018.07.030.

62. Jones, G.; Morrissette, P.J. Foster parent stress. *Can. J. Couns.* **1999**, *33*, 13–27.

63. Khoo, E.; Skoog, V. The road to placement breakdown: Foster parents' experiences of the events surrounding the unexpected ending of a child's placement in their care. *Qual. Soc. Work Res. Pract.* **2014**, *13*, 255–269, doi:10.1177/1473325012474017.

64. Kidman, R.; Thurman, T.R. Caregiver burden among adults caring for orphaned children in rural South Africa. *Vulnerable Child. Youth Stud.* **2014**, *9*, 234–246, doi:10.1080/17450128.2013.871379.

65. King, K.A.; Kraemer, L.K.; Bernard, A.L.; Vidourek, R.A. Foster parents' involvement in authoritative parenting and interest in future parenting training. *J. Child Fam. Stud.* **2007**, *16*, 606–614, doi:10.1007/s10826-006-9110-5.

66. Klee, L.; Kronstadt, D.; Zlotnick, C. Foster care's youngest: A preliminary report. *Am. J. Orthopsychiatry* **1997**, *67*, 290–299, doi:10.1037/h0080232.

67. Koren-Karie, N.; Markman-Gefen, R. Foster caregiver insightfulness and emotional investment in foster children. *J. Soc. Work* **2016**, *16*, 489–509, doi:10.1177/1468017315581528.

68. Krčar, M.; Laklija, M. Foster care from the perspective of Roma foster parents in Roma settlements in Međimurje county. *Kriminologija Soc. Integr.* **2018**, *26*, 183–207, doi:10.31299/ksi.26.2.3.

69. Landy, S.; Munro, S. Shared parenting: Assessing the success of a foster parent program aimed at family reunification. *Child Abus. Negl.* **1998**, *22*, 305–318, doi:10.1016/S0145-2134(97)00177-4.

70. Lanigan, J.D.; Burleson, E. Foster parent's perspectives regarding the transition of a new placement into their home: An exploratory study. *J. Child Fam. Stud.* **2017**, *26*, 905–915, doi:10.1007/s10826-016-0597-0.

71. Leake, R.; Wood, V.F.; Bussey, M.; Strolin-Goltzman, J. Factors influencing caregiver strain among foster, kin, and adoptive parents. *J. Public Child Welf.* **2019**, *13*, 285–306, doi:10.1080/15548732.2019.1603131.

72. Leathers, S.J.; Spielfogel, J.E.; Geiger, J.; Barnett, J.; Vande Voort, B.L. Placement disruption in foster care: Children's behavior, foster parent support, and parenting experiences. *Child Abuse Negl.* **2019**, *91*, 147–159, doi:10.1016/j.chiabu.2019.03.012.

73. Lindhiem, O.; Dozier, M. Caregiver commitment to foster children: The role of child behavior. *Child Abuse Negl.* **2007**, *31*, 361–374, doi:10.1016/j.chiabu.2006.12.003.

74. Lipscombe, J.; Farmer, E.; Moyers, S. Parenting fostered adolescents: Skills and strategies. *Child Fam. Soc. Work* **2003**, *8*, 243–255, doi:10.1046/j.1365-2206.2003.00294.x.

75. Lipscombe, J.; Moyers, S.; Farmer, E. What changes in "parenting" approaches occur over the course of adolescent foster care placements? *Child Fam. Soc. Work* **2004**, *9*, 347–357, doi:10.1111/j.1365-2206.2004.00343.x.

76. Lipton, M. The effect of the primary caretaker's distress on the sexually abused child: A comparison of biological and foster parents. *Child Adolesc. Soc. Work J.* **1997**, *14*, 115–127, doi:10.1023/A:1024505216535.

77. Lotty, M.; Bantry-White, E.; Dunn-Galvin, A. The experiences of foster carers and facilitators of Fostering Connections: The Trauma-informed Foster Care Program: A process study. *Child. Youth Serv. Rev.* **2020**, *119*, doi:10.1016/j.childyouth.2020.105516.

78. Maaskant, A.M.; van Rooij, F.B.; Overbeek, G.J.; Oort, F.J.; Hermanns, J.M.A. Parent training in foster families with children with behavior problems: Follow-up results from a randomized controlled trial. *Child. Youth Serv. Rev.* **2016**, *70*, 84–94, doi:10.1016/j.childyouth.2016.09.005.

79. McKeough, A.; Bear, K.; Jones, C.; Thompson, D.; Kelly, P.J.; Campbell, L.E. Foster carer stress and satisfaction: An investigation of organisational, psychological and placement factors. *Child. Youth Serv. Rev.* **2017**, *76*, 10–19, doi:10.1016/j.childyouth.2017.02.002.

80. McSherry, D.; Malet, M.F.; Weatherall, K. The Strengths and Difficulties Questionnaire (SDQ): A proxy measure of parenting stress. *Br. J. Soc. Work* **2019**, *49*, 96–115, doi:10.1093/bjsw/bcy021.

81. Miller, L.; Randle, M.; Dolnicar, S. Carer factors associated with foster-placement success and breakdown. *Br. J. Soc. Work* **2019**, *49*, 503–522, doi:10.1093/bjsw/bcy059.

82. Morrissette, P.J. Foster parenting: A developmental model. *Child Adolesc. Soc. Work J.* **1994**, *11*, 235–246, doi:10.1007/BF01875874.

83. Nesmith, A. False allegations and caseworker conflict: Stressors among long-term foster parents. *Child. Youth Serv. Rev.* **2020**, *118*, doi:10.1016/j.childyouth.2020.105435.

84. Orme, J.G.; Combs-Orme, T. Foster parenting together: Foster parent couples. *Child. Youth Serv. Rev.* **2014**, *36*, 124–132, doi:10.1016/j.childyouth.2013.11.017.

85. Perry, K.J.; Price, J.M. Concurrent child history and contextual predictors of children's internalizing and externalizing behavior problems in foster care. *Child. Youth Serv. Rev.* **2018**, *84*, 125–136, doi:10.1016/j.childyouth.2017.11.016.

86. Petrenko, C.L.M.; Alto, M.E.; Hart, A.R.; Freeze, S.M.; Cole, L.L. "I'm Doing My Part, I Just Need Help From the Community": Intervention Implications of Foster and Adoptive Parents' Experiences Raising Children and Young Adults With FASD. *J. Fam. Nurs.* **2019**, *25*, 314–347, doi:10.1177/1074840719847185.

87. Price, J.M.; Roesch, S.; Walsh, N.E.; Landsverk, J. Effects of the KEEP Foster Parent Intervention on Child and Sibling Behavior Problems and Parental Stress During a Randomized Implementation Trial. *Prev. Sci.* **2015**, *16*, 685–695, doi:10.1007/s11121-014-0532-9.

88. Rhodes, K.W.; Orme, J.G.; Cox, M.E.; Buehler, C. Foster family resources, psychosocial functioning, and retention. *Soc. Work Res.* **2003**, *27*, 135–150, doi:10.1093/swr/27.3.135.

89. Richardson, E.W.; Futris, T.G.; Mallette, J.K. Fostering confidence: Foster caregivers' relationship efficacy and associations with marital and co-parenting relationship quality. *Child Fam. Soc. Work* **2020**, *25*, 62–73, doi:10.1111/cfs.12654.

90. Sloan Donachy, G. The caregiving relationship under stress: Foster carers' experience of loss of the sense of self. *J. Child Psychother.* **2017**, *43*, 223–242, doi:10.1080/0075417X.2017.1323943.

91. Soliday, E.; McCluskey-Fawcett, K.; Meck, N. Foster mothers' stress, coping, and social support in parenting drug-exposed and other at-risk toddlers. *Child. Heal. Care* **1994**, *23*, 15–32, doi:10.1207/s15326888chc2301_2.

92. Tan, C.; Zhao, C.; Dou, Y.; Duan, X.; Shi, H.; Wang, X.; Huang, X.; Zhang, J. Caregivers' depressive symptoms and social–emotional development of left-behind children under 3 years old in poor rural China: The mediating role of home environment. *Child. Youth Serv. Rev.* **2020**, *116*, doi:10.1016/j.childyouth.2020.105109.

93. Unterberg, A.; Schröder, M.; Pérez, T.; Di Gallo, A.; Schmid, M. Der Zusammenhang von elterlichem Stress, Bindungsproblemen und psychischer Belastung von Pflegekindern = Relationship between parental stress, relationship problems, and mental stress among foster children. *Fam. Syst. Prax. und Forsch.* **2013**, *38*, 278–288.

94. Van Andel, H.W.H.; Post, W.J.; Jansen, L.M.C.; Kamphuis, J.S.; Van der Gaag, R.J.; Knorth, E.J.; Grietens, H. The developing relationship between recently placed foster infants and toddlers and their foster carers: Do demographic factors, placement characteristics and biological stress markers matter? *Child. Youth Serv. Rev.* **2015**, *58*, 219–226, doi:10.1016/j.childyouth.2015.10.003.

95. Van Holen, F.; Vanderfaeillie, J.; Omer, H.; Vanschoonlandt, F. Training in nonviolent resistance for foster parents: A randomized controlled trial. *Res. Soc. Work Pract.* **2018**, *28*, 931–942, doi:10.1177/1049731516662915.

96. Van Holen, F.; Vanschoonlandt, F.; Vanderfaeillie, J. Evaluation of a foster parent intervention for foster children with externalizing problem behaviour. *Child Fam. Soc. Work* **2017**, *22*, 1216–1226, doi:10.1111/cfs.12338.

97. van Rooij, F.B.; van der Put, C.; Maaskant, A.M.; Folkeringa, D.; Hermanns, J.M.A. Risk assessment for foster placement breakdown: The predictive value of the strengths and difficulties questionnaire and foster child and foster family characteristics. *Child. Youth Serv. Rev.* **2019**, *100*, 353–361, doi:10.1016/j.childyouth.2019.02.038.

98. Vanderfaeillie, J.; van Holen, F.; Trogh, L.; Andries, C. The impact of foster children's behavioural problems on Flemish foster mothers' parenting behaviour. *Child Fam. Soc. Work* **2012**, *17*, 34–42, doi:10.1111/j.1365-2206.2011.00770.x.

99. Vanderfaeillie, J.; Van Holen, F.; Vanschoonlandt, F.; Robberechts, M.; Stroobants, T. Children placed in long-term family foster care: A longitudinal study into the development of problem behavior and associated factors. *Child. Youth Serv. Rev.* **2013**, *35*, 587–593, doi:10.1016/j.childyouth.2012.12.012.

100. Vanschoonlandt, F.; Van Holen, F.; Vanderfaeillie, J.; De Maeyer, S.; Andries, C. Flemish foster mothers' perceptions of support needs regarding difficult behaviors of their foster child and their own parental approach. *Child Adolesc. Soc. Work J.* **2014**, *31*, 71–86, doi:10.1007/s10560-013-0310-8.

101. Vanschoonlandt, F.; Vanderfaeillie, J.; Van Holen, F.; De Maeyer, S.; Robberechts, M. Parenting stress and parenting behavior among foster mothers of foster children with externalizing problems. *Child. Youth Serv. Rev.* **2013**, *35*, 1742–1750, doi:10.1016/j.childyouth.2013.07.012.

102. Vasileva, M.; Petermann, F. Posttraumatic Stress Symptoms in Preschool Children in Foster Care: The Influence of Placement and Foster Family Environment. *J. Trauma. Stress* **2017**, *30*, 472–481, doi:10.1002/jts.22217.

103. Whenan, R.; Oxlad, M.; Lushington, K. Factors associated with foster carer well-being, satisfaction and intention to continue providing out-of-home care. *Child. Youth Serv. Rev.* **2009**, *31*, 752–760, doi:10.1016/j.childyouth.2009.02.001.

104. Whitt-Woosley, A.; Sprang, G.; Eslinger, J. Exploration of factors associated with secondary traumatic stress in foster parents. *Child. Youth Serv. Rev.* **2020**, *118*, doi:10.1016/j.childyouth.2020.105361.

105. Wilson, K.; Sinclair, I.; Gibbs, I. The trouble with foster care: The impact of stressful "events" on foster carers. *Br. J. Soc. Work* **2000**, *30*, 193–209, doi:10.1093/bjsw/30.2.193.

106. Cooley, M.E.; Womack, B.; Rush, J.; Slinskey, K. Adverse childhood experiences among foster parents: Prevalence and association with resilience, coping, satisfaction as a foster parent, and intent to continue fostering. *Child. Youth Serv. Rev.* **2020**, *109*, doi:10.1016/j.childyouth.2019.104679.

107. Fuentes, M.J.; Salas, M.D.; Bernedo, I.M.; García-Martín, M.A. Impact of the parenting style of foster parents on the behaviour problems of foster children. *Child. Care. Health Dev.* **2015**, *41*, 704–711, doi:10.1111/cch.12215.

108. García-Martín, M.A.; Salas, M.D.; Bernedo, I.M.; Fuentes, M.J. Foster care profiles: A guide to identifying at-risk placements. *J. Child Fam. Stud.* **2015**, *24*, 2579–2588, doi:10.1007/s10826-014-0060-z.

109. Lohaus, A.; Chodura, S.; Möller, C.; Symanzik, T.; Ehrenberg, D.; Job, A.-K.; Reindl, V.; Konrad, K.; Heinrichs, N. Children's mental health problems and their relation to parental stress in foster mothers and fathers. *Child Adolesc.*

Psychiatry Ment. Health **2017**, *11*, doi:10.1186/s13034-017-0180-5.

110. Megahead, H.A.; Deater-Deckard, K. Parenting stress and foster children's adjustment in an Egyptian context. *J. Child Fam. Stud.* **2017**, *26*, 2266–2275, doi:10.1007/s10826-017-0736-2.

111. Mennen, F.E.; Trickett, P.K. Parenting Attitudes, Family Environments, Depression, and Anxiety in Caregivers of Maltreated Children. *Fam. Relations An Interdiscip. J. Appl. Fam. Stud.* **2011**, *60*, 259–271, doi:https://dx.doi.org/10.1111%2Fj.1741-3729.2011.00646.x.

112. Miller, J.J.; Cooley, M.E.; Mihalec-Adkins, B.P. Examining the Impact of COVID-19 on Parental Stress: A Study of Foster Parents. *Child Adolesc. Social Work J.* **2020**, 1–10, doi:10.1007/s10560-020-00725-w.

113. Murray, L.; Tarren-Sweeney, M.; France, K. Foster carer perceptions of support and training in the context of high burden of care. *Child Fam. Soc. Work* **2011**, *16*, 149–158, doi:10.1111/j.1365-2206.2010.00722.x.

114. Richardson, E.W.; Futris, T.G.; Mallette, J.K.; Campbell, A. Foster mothers' parenting stress and coparenting quality: An examination of the moderating role of support. *Child. Youth Serv. Rev.* **2018**, *89*, 77–82, doi:10.1016/j.childyouth.2018.04.024.

115. Richardson, E.W.; Futris, T.G. Foster caregivers' marital and coparenting relationship experiences: A dyadic perspective. *Fam. Relations An Interdiscip. J. Appl. Fam. Stud.* **2019**, *68*, 185–196, doi:10.1111/fare.12354.

116. Gabler, S.; Bovenschen, I.; Lang, K.; Zimmermann, J.; Nowacki, K.; Kliewer, J.; Spangler, G. Foster children's attachment security and behavior problems in the first six months of placement: associations with foster parents' stress and sensitivity. *Attach. Hum. Dev.* **2014**, *16*, 479–498, doi:10.1080/14616734.2014.911757.

117. Gabler, S.; Kungl, M.; Bovenschen, I.; Lang, K.; Zimmermann, J.; Nowacki, K.; Kliewer-Neumann, J.; Spangler, G. Predictors of foster parents' stress and associations to sensitivity in the first year after placement. *Child Abuse Negl.* **2018**, *79*, 325–338, doi:10.1016/j.chiabu.2018.02.009.

118. Bergsund, H.B.; Wentzel-Larsen, T.; Jacobsen, H. [Parenting stress in long-term foster carers: A longitudinal study]. *Child & Fam. Soc. Work* **2020**, *25*, 53–62, doi:10.1111/cfs.12713.

119. Lohaus, A.; Kerkhoff, D.; Chodura, S.; Möller, C.; Symanzik, T.; Rueth, J.E.; Ehrenberg, D.; Job, A.-K.; Reindl, V.; Konrad, K.; et al. Longitudinal relationships between foster children's mental health problems and parental stress in foster mothers and fathers. *Eur. J. Heal. Psychol.* **2018**, *25*, 33–42, doi:10.1027/2512-8442/a000007.
